# PLAN-M; Mycobacteriophage Endolysins Fused to Biodegradable Nanobeads Mitigate Mycobacterial Growth in Liquid and on Surfaces

**DOI:** 10.3389/fmicb.2021.562748

**Published:** 2021-04-26

**Authors:** Courtney G. Davies, Kerri Reilly, Eric Altermann, Heather L. Hendrickson

**Affiliations:** ^1^Massey Phage Whānau, School of Natural and Computational Sciences, Massey University, Auckland, New Zealand; ^2^AgResearch Ltd., Palmerston North, New Zealand; ^3^Riddet Institute, Massey University, Palmerston North, New Zealand; ^4^Infectious Disease Research Centre, Massey University, Palmerston North, New Zealand

**Keywords:** Lysin A, Lysin B, personal protective equipment, polyhydroxyalkanoates, PHA beads

## Abstract

The Mycobacteria are a genus of Actinobacteria that include human pathogens such as *Mycobacterium tuberculosis* (TB). Active TB disease can spread by airborne transmission to healthcare workers and to their community. The HHMI SEA-PHAGES program has contributed to discovering bacteriophages that are able to infect *M. smegmatis* MC^2^ 155, a close relative of *M. tuberculosis*. This collection of diverse Mycobacteriophages is an excellent resource for trialling bacteriophage-sourced enzymes in novel applications. Herein we measured the ability Mycobacteriophage endolysins to lyse their host strain when functionally fused to biodegradable polyhydroxyalkanoate (PHA) nanobeads. PHA nanobeads facilitate both the expression and the application of enzymes to surfaces and have been demonstrated to stabilize a wide array of proteins for practical applications whilst eliminating the challenges of traditional protein purification. We selected two Lysin A and six Lysin B homologs to be functionally fused to the polyhydroxyalkanoate synthase C (PhaC). Expression of these constructs resulted in functional lysins displayed on the surface of PHA nanobeads. The lysins thus directionally displayed on nanobeads lysed up to 79% of the *M. smegmatis* MC^2^ 155 population using 80 mg/mL of nanobeads in pure culture. In order to determine whether the nanobeads would be effective as a protective layer in PPE we adapted a fabric-based test and observed a maximum of 1 log loss of the cell population after 5 h of exposure on a textile (91% cell lysis). Lysin B enzymes performed better than the Lysin A enzymes as a protective barrier on textiles surface assays. These results suggest that bacterial endolysins are efficient in their action when displayed on PHA nanobeads and can cause significant population mortality in as little as 45 min. Our results provide the proof-of-principle that Mycobacteriophage endolysins can be used on functionalized nanobeads where they can protect surfaces such as personal protective equipment (PPE) that routinely come into contact with aerosolised bacteria.

## Introduction

The genus Mycobacterium includes over 150 recognized species, many of which are human pathogens (King et al., 2017). Of these, *M. tuberculosis* (TB) is among the most serious; one-fourth of the global population is estimated to carry latent tuberculosis infection (Houben and Dodd, 2016). In 2017, the WHO reported that active tuberculosis infections caused 1.3 million deaths, 558,000 people developed TB infections that were resistant to the frontline drug rifampicin, and 82% of reported cases were resistant to two of the most powerful anti-tuberculosis drugs (WHO, 2019). Once infected, an individual can transmit tuberculosis to their community through aerosolised bacteria expelled in droplets from the lungs (Hannan et al., 2000). Airborne transmission is of special concern in clinical settings, where health care workers exposed to coughing patients are at high risk of infection (Escombe et al., 2007; Ruhl et al., 2020). Mitigating the risk of infection and safeguarding healthcare professionals and the community with bespoke Personal Protective Equipment (PPE) is therefore a high priority.

Bacteriophages (phages) are viruses that parasitize bacteria and they are the most abundant entities on earth (Abedon, 2008). The global increase in antibiotic-resistant pathogens has renewed interest in the potential of bacteriophages, bacteriophage-based therapies, and phage-based products to combat antibiotic-resistant pathogens. Bacteriophages encode lysins that are released at the end of the infection cycle in order to allow the viruses to destroy the cell wall from within and propagate further by host cell rupture (Young, 2014). This is accomplished through the coordinated action of a Holin protein or proteins, which form pores in the cell membrane and facilitate the transit of endolysins across the inner membrane to access the cell wall. Endolysins are a diverse class of enzymes and can be discovered in cultured and uncultured bacteriophage genomes (Fernández-Ruiz et al., 2018). Half of the global population of bacteria are turned over by bacteriophage mediated lysis every 48 h, making endolysins the most effective and widespread bactericidal agent on the planet (Fischetti, 2018).

**FIGURE 1:**
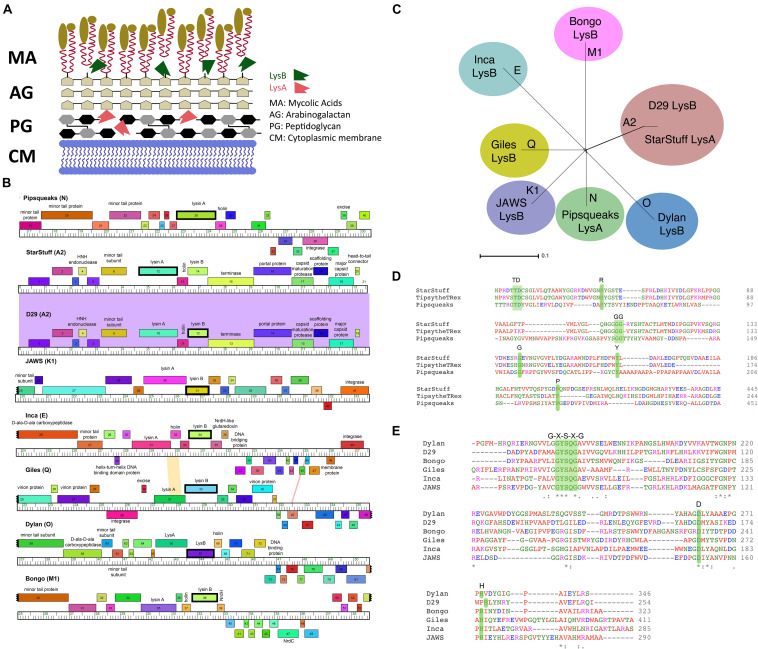
Selecting LysA and LysB endolysins for PLAN-M nanobeads. **(A)** The structure of a typical *Mycobacterium* cell wall and the points of Lysin A (pink), a glycoside hydrolase acting on the peptidoglycan cell wall and Lysin B (green) a lipolytic enzyme disrupting the bonds between the mycolic acids and the arabinogalactan layer. Tan ovoids represent a diverse set of lipids and enzymes that can be attached to the Mycolic acids (adapted from Gerstmans et al., 2016). **(B)** Phamerator map of genetic regions for each of the mycobacteriophages endolysins (in bold) being used in this study. The colored regions between phage genomes indicate levels of sequence homology (purple indicates high homology). **(C)** A SplitsTree representation of the selected bacteriophage genomes with their respective clusters labeled (in bold) shows no evidence of phylogenetic ambiguity or gene transfer between the selected bacteriophage clusters. **(D)** The conserved residues predicted to be important for function in the Lysin A and **(E)** Lysin B proteins in this study. Green shaded regions in amino acid comparisons are key functional amino acid residues that are conserved in our chosen sequences.

There are two major obstacles to the release of bacteriophage particles from mycobacterial cells, the peptidoglycan cell wall and the mycolylarabinogalactan layer. Mycobacteriophages often encode separate endolysins to overcome these cell structures in turn. Lysin A, encoded by *lysA* genes, are peptidoglycan hydrolyzing enzymes that act directly on the peptidoglycan cell wall (CW) (Figure 1A). Lysin A proteins generally contain an N-acetyl-β-D-muramidase domain of the glycoside hydrolase family. Mycobacteriophages often carry a second endolysin, the Lysin B protein, encoded by *lysB* genes. Lysin B enzymes have lipolytic activity and disrupt mycobacterial integrity by cleaving the bonds between the arabinogalactan layer and the outer mycolic acids (Figure 1A; Gil et al., 2010; Payne and Hatfull, 2012).

Phage encoded lysins of Gram-positive bacteria bring about “lysis from without,” destroying cells when applied extracellularly (Abedon, 2011). This is in part because the cell wall of Gram-positive bacteria is accessible from without whilst the cell wall of Gram-negative bacteria is much less accessible (Loessner, 2005). The first demonstration of the safe application of a lysin in a therapeutic approach was demonstrated in *Streptococcus* using the C_1_ phage lysin (Nelson et al., 2001). Lysin C_1_ had previously proved difficult to isolate in an active form due to inactivation of the lysin activity during column-based purification (Fischetti, 2018). Lysin mediated lysis from without has been demonstrated for a wide range of Gram-positive bacteria including *Listeria, Bacillus, Clostridium*, and *Streptococcus* (Loessner, 2005).

*M. smegmatis* MC^2^ 155, is a non-pathogenic relative of *M. tuberculosis* and widely used as a model organism. One of the early examples of a Mycobacteriophage was *M. smegmatis* MC^2^ 155 phage D29 (Ford et al., 1998). Mycobacteriophage D29 is able to infect *M. tuberculosis*, making it an attractive phage for potential therapeutics (Bavda and Jain, 2020) and it has been used prophylactically to prevent *M. tuberculosis* H37Rv infection in a mouse model (Carrigy et al., 2019). Further work has demonstrated that Lysin B from D29 can be used prophylactically to treat *M. ulcerans* infections in a mammalian ulcer assay (Fraga et al., 2019).

The extracellular application of purified Lysin B enzyme from Mycobacteriophage D29 has been shown to cause lysis from without and to disrupt biofilms (Payne et al., 2009). Lysin B from phage D29 is a mycolylarabinogalactan esterase which contains an α/β hydrolase, a catalytic triad common to cutinases, and an additional four-helix domain involved in binding lipid substrates (Payne et al., 2009). It acts by cleaving the specific ester linking the mycolic acid-rich outer membrane to the arabinogalactan, releasing free mycolic acids and causing cell lysis (Payne et al., 2009).

Our knowledge of *M. smegmatis* MC^2^ 155 specific bacteriophages has benefited from the collective efforts of the student phage hunters in the Howard Hughes Medical Institute’s Science Education Alliance-Phage Hunters Advancing Genomics and Evolutionary Science (SEA-PHAGES) program (Jacobs-Sera et al., 2012; Cross et al., 2013). The discovery efforts of the SEA-PHAGES program have resulted in the largest collection of bacteriophages for a single host strain (phagesdb.org, 2020).

Polyhydroxyalkanoates (PHA) are the largest group of natural biopolymers, produced in various microorganisms as carbon and energy reserves when nutrients are limited (Kamravamanesh et al., 2018). In nature, PHA polymers are carbon storage reserves that are naturally degraded by organisms possessing the PHA depolymerase PhaZ (Chek et al., 2017). PHA nanobeads have been developed for use in biomedical and industrial applications, have been shown to be well tolerated by mammalian systems and are water-insoluble and biodegradable (Peters and Rehm, 2005, 2006; Grage et al., 2009).

PHA nanobeads consist of hydroxyalkanoic acids linked by oxoester bonds with an amorphous polyester core surrounded by a boundary layer of embedded or attached proteins (Li et al., 2016). PHA nanobeads are synthesized *in vivo* by the PHA synthase PhaC which remains covalently bound to its own polymer molecule. Through the hydrophobic nature of PHA polymers, PHA nanobeads form spontaneously within the cell and accumulate to up to 80% of the dry cell weight, ultimately ranging in size from 100 to 500 nm (Rehm, 2003; Grage et al., 2009). PhaC can tolerate both N- and C-terminal protein fusions without losing activity, a feature that has been utilized to fuse a range of enzymes to PhaC to be directionally displayed on the surface of PHA nanobeads in a one-step biosynthesis (Altermann et al., 2018).

PHA nanobeads can be synthesized in *Escherichia coli* and subsequent cell lysis and bead purification results in tailored nanoparticles ready to be used in a biotechnical or biomedical applications (Blatchford et al., 2012). PHA nanobeads offer three distinct advantages to other possible expression methods: (1) proteins of interest are covalently bound and stabilized in a uniform direction to the surface of the nanobead, (2) the stabilizing matrix of the nanobeads enables ready deployment of proteins and enzymes in liquids or on surfaces, and (3) these can be expressed in a one-step process (Lee et al., 2017).

Herein we describe the selection, manufacture and validation of a set of eight Mycobacteriophage endolysins fused to PHA nanobeads along with an endolysin-free negative control. We hypothesize that tailored nanobeads will present a novel protective layer capable of reducing live bacterial carriage in liquid and on a textile fabric. Based on previous studies, we further hypothesize that Lysin B will be more effective at reducing bacterial carriage than Lysin A in this framework.

## Materials and Methods

### Selecting Endolysins for Analysis

PhagesDB (phagesdb.org, 2020) is a database that contains details of over 17,750 Actinobacteriophages, of which 1,880 + infect *Mycobacterium* species and are fully sequenced and annotated (Russell and Hatfull, 2017). This resource is maintained as a comprehensive open-access database that collates bacteriophages that have been discovered in the SEA-PHAGES program and related programs (Russell and Hatfull, 2017).

A set of sequenced mycobacteriophages that are known to infect *M. smegmatis* MC^2^ 155 were selected from PhagesDB (Russell and Hatfull, 2017). Each endolysin amino acid sequence was subjected to an NCBI Standard Protein BLASTP alignment to verify the annotated function by sequence similarity to other endolysins (Goujon et al., 2010; Sievers et al., 2011).

In order to further validate the functional identity of *lysA* and *lysB* homologs, amino acid sequences were submitted as FASTA files to the Max Planck Institute (MPI) Bioinformatics Toolbox HHpred (NCBI_Conserved_Domains (CD)_v3.16) (Zimmermann et al., 2018). The top matches were recorded and compared to known endolysin results in order to evaluate the validity of the annotation calls in the published genome sequences (Goujon et al., 2010; McWilliam et al., 2013).

In order to determine the degree to which the eight bacteriophages are distinct, Phamerator (Cresawn et al., 2011) was used to visualize the genomes. A tree showing the distinctiveness of these bacteriophages, and their assigned clusters (phagesdb.org, 2020) was constructed using SplitsTree (Kloepper and Huson, 2008).

### Media and Molecular Methods

The following antibiotics were used as appropriate: ampicillin (AMP) 50 μg/mL, chloramphenicol (CM) 34 μg/mL, carbenicillin disodium salt (CB) 50 μg/mL and cycloheximide (CHX) 10 μg/mL. *M. smegmatis* MC^2^ 155 was streaked out from frozen stocks onto 1.5% Agar Lysogeny Broth (LB) plates + CB + CHX and incubated at 37°C for 72 h. *M. smegmatis* MC^2^ 155 was propagated from a single colony in complete Middlebrook 7H9 liquid media supplemented with 0.2% glycerol and 0.05% Tween80 at 37°C, shaking at 250 rpm for 72 h. CaCl_2_ was added at a final concentration of 1 mM and Tween80 was omitted when bacteriophages were in use. Working stocks were stored at room temperature and active *M. smegmatis* MC^2^ 155 cultures were produced by subculturing these stocks in Middlebrook 7H9 liquid media as above at 37°C for 72 h. *E. coli* BL21 cells were grown in LB liquid media at 37°C with shaking, adding antibiotics as appropriate.

### Producing PLAN-M Nanobeads

Standard methods were used to transform chemically competent *E. coli* BL21 cells with the pMCS69 helper plasmid which harbors the *phaA* and *phaB* genes, required to synthesize PHA precursors (González-Miró et al., 2018). The endolysin-*phaC* gene fusion was designed and then subsequently codon optimized, mRNA stabilized and synthesized by GeneArt (Thermo Fisher Scientific, GENEART GmbH, Regensburg, Germany). The synthetic genes were subcloned into the pET14b protein expression vector under control of the LacZ promoter (González-Miró et al., 2018; Supplementary Figure 1A). The complete sequences for these cloned synthetic vectors are available at NCBI under their respective accession numbers (Table 1).

**TABLE 1:** The bacteriophages used in this study, including the locations and types of lysins selected from each bacteriophage, key features and NCBI accession numbers for the codon adapted sequences of each lysin.

Bacteriophage (cluster)	Gene number	Lysin	Vector name	Key features	NCBI accession
Bongo (Ml)	38	Lysin B	pET14b_17AECEAC_Bongo-L-PhaC	(Bongo) *LysB-PhaC*	MW151033
D29 (A2)	12	Lysin B	pET14b_17AECD4C_D29-L-PhaC	(D29) *LysB-PhaC*	MW151034
Dylan (0)	67	Lysin B	pET14b_17AECD7C_Dylan-L-PhaC	(Dylan) *LysB-PhaC*	MW151035
Giles (Q)	32	Lysin B	pET14b_17AECEBC_Giles-L-PhaC	(Giles) *LysB-PhaC*	MW151036
Inca (E)	34	Lysin B	pET14b_17AECD6C_lnca-L-PhaC	(Inca) *LysB-PhaC*	MW151037
JAWS (Kl)	31	Lysin B	pET14b_17AECD5C_JAWS-L-PhaC	(JAWS) *LysB-PhaC*	MW151038
Pipsqueaks (N)	28	Lysin A	pET14b_17AECD3C_Pipsqueaks-L-PhaC	(Pipsqueaks) *LysA-PhaC*	MW151039
StarStuff (A2)	12	Lysin A	pET14b_17AECD2C_StarStuff-L-PhaC	(StarStuff) *LysA-PhaC*	MW151040

An overview of the process of producing the nanobeads is shown (Supplementary Figure 1B). Chemically competent *E. coli* BL21 + pMCS69 cells were transformed with the endolysin-*phaC* fusion gene harboring pET14b-based plasmid, and selected on LB + AMP + CM plates incubated at 37°C. *E. coli* BL21 (pMCS69 + PhaC pET14b) plasmids were incubated overnight in a shaking incubator in 5 mL LB + AMP + CM at 37°C. One milliliter of these cultures were added to 500 mL flasks with 100 mL fresh LB + AMP + CM and 20% glucose.

Cultures were incubated at 37°C, shaking at 200 rpm for 2 h or until they reached an OD600 of 0.5. One milliliter of 100 mM IPTG was added to each flask to induce expression of the PhaC-Lysin fusions on the pET-14b plasmid, which initiated the production of the PHA beads inside the *E. coli* cells (Abedi et al., 2012). Induced cultures were incubated at 20°C shaking at 160 rpm for 48 h. After incubation, cells were centrifuged at 5,000 g for 10 min. The supernatant was then discarded and the pellet was frozen until the bacterial lysis procedure commenced.

In order to lyse *E. coli* cells, the bacterial pellet was resuspended in 10 mL of lysis buffer [50 mM Tris pH 7.5 containing 2 mM DTT, 300 mM NaCl, 10 mM imidazole, 1% Triton X-100 (v/v), 20% glycerol (v/v)] lysozyme (10 mg/mL) and 150 μL of DNase (1U/μL) at 4°C overnight, after which the solution was brought to a total volume of 40 mL with the addition of Phosphate Buffered Saline (PBS) and vortexed before being placed on ice. Cells were lysed one of two ways depending on the facility in which the PLAN-M nanobeads were being produced. They were either sonicated using the Misonix Sonicator Ultrasonic Processor S-4000 (Farmingdale, NY, United States) for additional physical cell lysis using an amplitude of 60, process time of 10 min, pulse-ON time; 30 s, pulse-OFF time 30 s for a total program of 5 min with intermittent shaking. Alternatively, cells were lysed using a Microfluidics (Westwood, MA) M-110PS microfluidizer. In this case, lysozyme treated samples were passed through a Diamond G10Z interaction chamber 10 times on ice to ensure complete lysis. These lysis methods were considered to be equivalent for the purpose of manufacturing the nanobeads.

After cell lysis, the remaining solution was centrifuged at 8,000 g for 20 min at 4°C. The supernatant was discarded and the pellet was resuspended with 4 mL of 50 mM phosphate buffer. A glycerol step-gradient with 4 mL of 88% glycerol on the base, 4 mL of 44% glycerol in the middle and 2 mL of resuspended pellets were made up in polypropylene tubes. The beads and glycerol gradients were centrifuged using an ultracentrifuge TW-641 rotor for 1 h 45 min at 35,000 RPM. After centrifugation, the white band containing the nanobeads at the glycerol gradient interface was removed and brought to a volume of 45 mL with PBS. This solution was shaken gently and centrifuged at 8,000 g for 20 min at 4°C to separate the purified nanobeads from any remaining glycerol. The supernatant was discarded, and the nanobead pellet was resuspended in phage buffer [10 mM Tris (pH 7.5), 10 mM MgSO4, and 68 mM NaCl] to a concentration of 20 mg/mL with the addition of 20 μL/mL Tween80.

Purified nanobead aliquots were stored at −80°C. Once in use, nanobeads were kept in the fridge at 4°C and not continuously frozen/refrozen. Nanobeads were produced at AgResearch in Palmerston North NZ and at Massey University in Albany NZ, using the protocol described. Imaging of PLAN-M nanobeads was achieved by freeze drying nanobeads at −80°C prior for 48 h prior to imaging under scanning electron microscopy at the Manawatu Microscopy and Imaging Centre, Massey University, Palmerston North New Zealand.

### Standing Culture Tests

Liquid culture nanobead exposures were performed in which PLAN-M nanobeads were added to 1 mL of standing culture of *M. smegmatis* MC^2^ 155 at 21°C for 45 min or 300 min in concentrations of either 10 or 80 mg/mL. Cultures were serially diluted in Middlebrook 7H9 Complete media, to a dilution of 10^–6^ and spread-plated on 1.5% agar LB + CB + CHX plates using autoclaved glass beads. Plates were inverted and incubated at 37°C for 72 h. After 72 h, colonies were counted and compared to both a no-nanobead control and a wild-type PhaC nanobead without a fused endolysin, to estimate the effective reduction in the cell population. The proportion of each population that had been lysed was estimated by first determining the number of surviving cells by CFU plating. The efficiency of lysis as a percent of surviving cells was determined for each experiment according to the equation:

$$Relativelysis\;[5]=(AverageCFU_{treatment}\text{)/}(AverageCFU_{negative\;control)}\text{)}\times \text{100}$$

Statistical analyses were subsequently conducted by pairwise *T*-tests to determine significance between nanobead controls (PhaC) and nanobead treatments (lysin-displayed nanobeads) (*N* = 3).

All experiments included a no nanobead control treatment which was used to estimate the average number of CFU expected in the absence of nanobeads. Blank nanobeads (those without lysins) and no nanobead treatment cultures were not significantly different indicating that nanobeads alone did not lyse *M. smegmatis* MC^2^ 155 in these experiments. Three biological replicates of each experiment were performed.

Combinations of Lysin A and Lysin B were assayed such that the total concentration of PLAN-M nanobeads applied to the bacterial culture was a 1:1 ratio of each. Otherwise, these experiments were as described above.

### The AATCC Fabric Test Method 100–2004

A standard industry test for antimicrobial effect of a treated fabric was performed to test the survival of *M. smegmatis* MC^2^ 155 after exposure to treated surfaces (American Association of Textile Chemists and Colorists, 2005). The AATCC 100–2004 method was modified slightly as follows to test the effectiveness of PLAN-M nanobead lysis. Filter paper was cut into three circular swatches of 4.8 cm diameter and autoclaved in aluminum before being placed in a sterile beaker. PLAN-M nanobeads were added to a final concentration of 80 mg/mL as appropriate and PBS was used for the negative control. 1 mL of *M. smegmatis* MC^2^ 155 overnight culture was added to each beaker and allowed to sit at room temperature for 45 or 300 min as appropriate. After the allotted time, 100 μL of the applied bacterial culture was diluted and plated on appropriate agar plates and allowed to incubate at 37°C for 72 h in order to estimate the CFU per mL remaining in the beaker. All PLAN-M nanobead exposures were tested for significance against these wild-type PhaC nanobeads to determine if the endolysins were affecting bacterial survival and *T*-tests were performed to evaluate the statistical significance of cell survival compared to the blank PhaC nanobead control. Blank nanobeads (those without lysins) and no nanobead treatment cultures were not significantly different indicating that nanobeads alone did not lyse *M. smegmatis* MC^2^ 155 in these experiments. Three biological replicates of each experiment were performed.

### Evaluating Resistance

A selection of *M. smegmatis* MC^2^ 155 colonies that had survived PLAN-M bead pure culture assay to grow on CFU plates were subsequently single colony isolated, grown in appropriate media and frozen at −80°C for further study. These were assayed for resistance by two methods. The first was to test them for resistance by using these as the pure cultures in the pure culture assay. The second method was the Spot Test PFU test to determine whether cultures of bacteria that had previously survived an exposure to a PLAN-M nanobead and formed CFU had developed an intrinsic resistance to a bacteriophage that uses one of these Lysin As. A spot test dilution was performed for test cultures derived from preserved surviving colonies. The culture of interest was plated in a top agar overlay and these were subjected to a serial dilution of bacteriophage Inca (Jacobs-Sera et al., 2012). A change in the maximum dilution at which phage plaques were still observed, relative to the WT bacteria (unexposed) was used to determine if any biologically relevant resistance to bacteriophage infection had occurred as a result of PLAN-M exposure.

## Results

### Selection of Bacteriophage Endolysins Used in This Study

A set of seven novel bacteriophages, chosen from separate Mycobacteriophage clusters, as defined by average nucleotide dissimilarity were chosen for this study (Hatfull et al., 2010). One of these seven was Mycobacteriophage Inca, a phage previously discovered in New Zealand as part of the SEA-PHAGES program (Davies et al., 2019). In addition, endolysin Lysin B from phage D29 was included as a known positive control that had previously been demonstrated to have the ability to lyse *M. smegmatis* MC^2^ 155 cells from without as a purified protein (Payne and Hatfull, 2012). The final selection of eight bacteriophage endolysins chosen, their clusters and respective gene numbers are listed in Table 1 (Ford et al., 1998; Pope et al., 2014; Butela et al., 2017; Dedrick et al., 2019). The genomic regions of these homologs are distinct with the exception of the two A2 cluster phages, StarStuff and D29. These bacteriophages are syntenic in this region but Lysin A was chosen from StarStuff whereas Lysin B was selected from D29 (Figure 1B).

These bacteriophages are genetically distinct and well isolated from one another, as demonstrated by a lack of inter-branch connections in a gene-content SplitsTree representation (Figure 1C). Together, these data suggest that the homologs selected are distinct from one another and represent a broad swath of the phylogenetic diversity observed in the Mycobacteriophages (Pope et al., 2015).

For each of these endolysins, predicted functions were confirmed using BLASTP (Payne et al., 2009; Payne and Hatfull, 2012). A non-redundant BLASTP search of each endolysin amino acid sequence against the non-redundant NCBI amino-acid database confirmed their best matches were consistent with the identity of Lysin A or Lysin B homologs, as annotated.

The identity of the lysins was further verified. The predicted amino acid sequence was subjected to an HHPred domain search. Each lysin returned best matches in HHpred that suggest that they function as expected. Lysin A homologs are peptidoglycan hydrolyzing enzymes and had high probability HHpred matches to chitinase_glyco_hydro_19 (a chitinase). Lysin A from phage Pipsqueaks had additional significant matches to a peptidase in a second region of the predicted protein, suggesting a second functional domain in this Lysin A enzyme. In addition, alignment of the amino acid sequences for these selected Lysin A homologs are consistent with previously described conserved motifs (Figure 1D; Payne and Hatfull, 2012). Taken together, these data support the assignment of Lysin A function to the *lysA* genes as annotated in StarStuff and Pipsqueaks.

Similarly, the Lysin B homologs had high probability HHpred matches to several lipases. This was consistent with previous work describing the Lysin B enzyme from D29 as having an α/β hydrolase activity (Payne et al., 2009). The lipases are believed to function at membranes as esterases that are able to hydrolyze long-chain acyl-triglycerides into their component parts (Figure 1A).

The Lysin B candidates demonstrated a conserved catalytic triad of specifically ordered Ser, Asp (or Glu) and His amino acid sequences, which had previously been identified in the 3D structure of D29 Lysin B. The G-X-S-X-G region is also conserved in all of the selected Lysin B homologs including the critical Ser amino acid position (Catalão and Pimentel, 2018; Figure 1E). Together, these data suggest that both the Lysin A and Lysin B homologs we selected are both distinct from one another and have been appropriately annotated.

**FIGURE 2:**
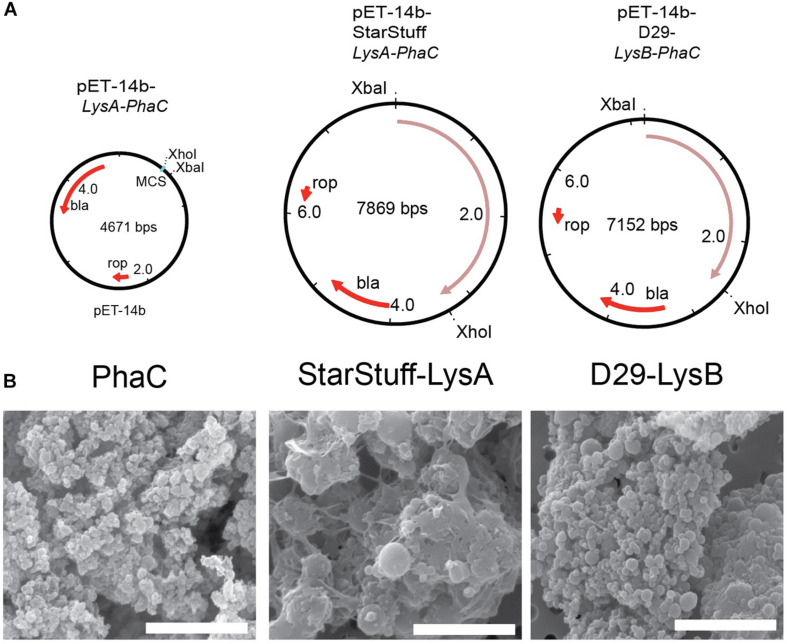
Synthesizing the PLAN-M nanobeads. **(A)** Genetic maps of the empty PhaC pET-14b vector, pET-14b StarStuff-LysA PhaC, and pET-14b-D29-LysB-PhaC, respectively. **(B)** Representative PLAN-M nanobeads produced from the vectors shown in **(A)** under 20,000x TEM. Scale bars are 3 nm.

The selected endolysin genes were codon optimized for expression in *E. coli* and functionally fused to the *phaC* gene in order to construct vectors that, when expressed will produce a nanoparticle with an N-terminal fusion of the Lysin As and Lysin Bs with PhaC, respectively. The subsequent synthesis of PHA polymer particles resulted in the formation of tailored PHA nanobeads with the lysin-PhaC fusion protein covalently displayed on the nanobead surface. The map of the empty PhaC vector in addition to two exemplars, StarStuff-LysA and D29-LysB are shown (Figure 2A).

One-hundred milliliter of transformed and cultured *E. coli* cells produced between 344.9 mg (Bongo-Lysin B) and 909.0 mg (Giles-Lysin B) of PLAN-M nanobeads (net weight). The nanobeads produced varied in size from 0.43 μm ± 0.015 (PhaC alone) to 1.78 μm ± 0.046 (StarStuff-Lysin A) in diameter, were generally spherical and homogeneous under Scanning Electron Microscopy (SEM) (Figure 2B), although StarStuff-Lysin A nanobeads appeared to be heterogeneous. The largest nanobeads were observed for the Lysin A of phage StarStuff and Pipsqueaks, approximately 2.5 times the size of the other nanobeads. The reason for this discrepancy in size is not known but may involve variations in bead processing during purification in *E. coli* or intrinsic properties of the endolysins (Altermann et al., 2018).

### PLAN-M Nanobeads Show Biological Activity Against Pure Cultures of *M. smegmatis* MC^2^ 155

In order to establish if the lysins displayed on PLAN-M nanobeads had lytic activity against *M. smegmatis* MC^2^ 155, we tested their ability to lyse *M. smegmatis* MC^2^ 155 cells through physical contact in liquid culture. We predicted that liquid conditions might allow the cells and beads to freely interact, readily exposing cells to the displayed endolysins.

**FIGURE 3:**
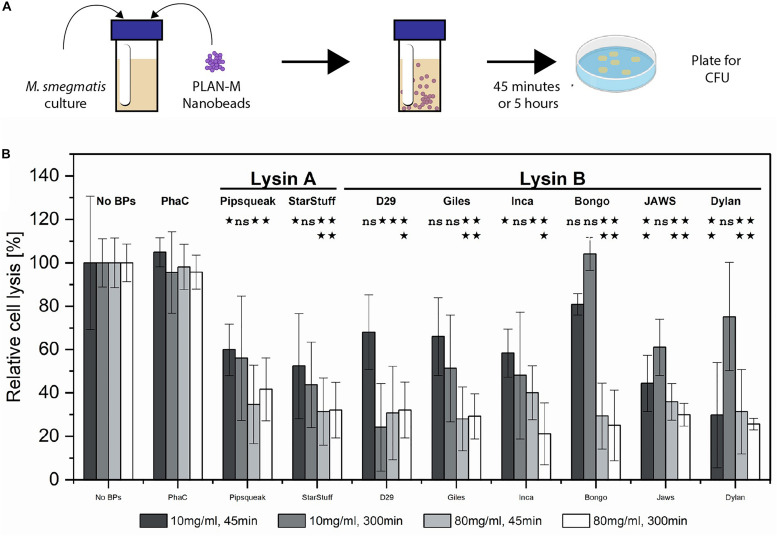
Pure culture test of biological activity of PLAN-M nanobeads against *M. smegmatis* MC^2^ 155. **(A)** Liquid overnight cultures of 1 mL of *M. smegmatis* MC^2^ 155 were subjected to 10 or 80 mg/mL concentrations of each of 9 PLAN-M nanobeads for 45 min or 5 h. After the allotted time these were plated for CFU. **(B)** Relative cell lysis of *M. smegmatis* MC^2^ 155 after treatment by lysin-free (PhaC) or PLAN-M nanobeads in pure culture tests (*n* = 3). Surviving populations are reported as percent change compared to the no nanobead control. Significance test results reported above the bars as *p-*values from Student’s *t*-test as follows; ns > 0.05, *< 0.05, **< 0.01, ***< 0.001. Error bars represent standard error based on three replicates of each experimental test.

*M. smegmatis* MC^2^ 155 cultures were subjected to nanobead concentrations of 10 mg/mL and 80 mg/mL for either 45 min or 5 h, respectively, and subsequently plated to enumerate CFU (Figure 3A). CFU in the presence and absence of nanobeads was used to calculate the efficiency of lysis (Figure 3B). Nanobeads without fused endolysins were included as negative controls and are shown (PhaC). A two-tailed Student’s *t*-test was calculated between each nanobead displaying lysin enzymes and the no lysin controls (PhaC), assuming equal variance to evaluate significance (Figure 3B). *P*-values above 0.05 were deemed insignificant (ns).

Forty-five minutes of exposure to 10 mg mL^–1^ of a single nanobead resulted in significant cell death in the *M. smegmatis* MC^2^ 155 populations in all PLAN-M nanobead treatments with the exception of the Lysin Bs of Bongo, Giles and D29, none of which were significantly distinct from the PhaC (Blank) treatment (Figure 3B, dark grey). However, prolonged exposure of 300 min (5 h) at 10 mg mL^–1^ was maintained in a separate experiment in which the *M. smegmatis* MC^2^ 155 cells did not demonstrate significant cell death compared to control beads with the exception of the Lysin B-D29 (Figure 3B medium gray).

When PLAN-M functionalized nanobeads were used at a higher concentration of 80 mg mL^–1^ for 45 min, timely and efficient lysis was observed, especially in Giles-Lysin B and Bongo-Lysin B with 29 and 25% cell survival, respectively (Figure 3B, light grey). The least effective of the PLAN-M nanobeads in this condition, however, still performed to promising levels. Inca-Lysin B induced cell death of 60% of the population after 45 min. In summary, all of these PLAN-M nanobeads were able to cause statistically significant reductions in the population of *M. smegmatis* at a concentration of 80 mg mL^–1^ after 45 min of pure culture standing liquid exposure.

Five hours of exposure to the 80 mg mL^–1^ concentration yielded similar results. Most of the PLAN-M nanobeads improved in their efficacy when they were present in higher concentrations and over either time period. Ultimately, in standing liquid cultures, PLAN-M beads with the Inca-Lysin B displayed had the highest average population lysis after 5 h with only 21% cell survival and the lowest average population lysis in this condition was PLAN-M displaying Pipsqueaks Lysin A at 41.6%, again suggesting a decrease in 60% of the population after 5 h of exposure in this condition (Figure 3B, white). Increasing the concentration of PLAN-M nanobeads to 80 mg mL^–1^ reduced the variability in *M. smegmatis* population survival in standing liquid cultures exposed for either 45 min or 5 h (Figure 3B, light grey and white).

**FIGURE 4:**
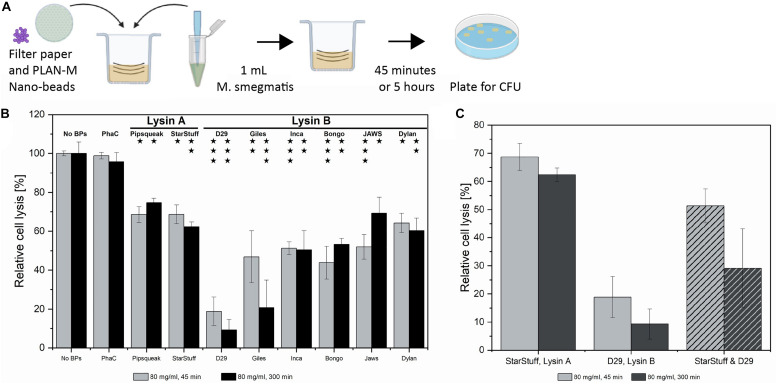
Textile test of PLAN-M nanobeads against *M. smegmatis* MC^2^ 155. **(A)** The AATCC-100-2004 protocol involves using a treated textile, in this case, filter papers with 80 mg/mL of each of 8 PLAN-M nanobeads. A 1 mL aliquot of *M. smegmatis* MC^2^ 155 culture was applied for exposures of 45 min and 5 h. **(B)** Relative cell lysis of *M. smegmatis* MC^2^ 155 after treatment by lysin-free (PhaC) or PLAN-M nanobeads (*n* = 3). **(C)** Relative cell lysis *M. smegmatis* MC^2^ 155 after treatment by two PLAN-M nanobeads; StarStuff-LysA and D29-LysB, in a 1:1 ratio and a final concentration of 80 mg/mL combined (40 mg/mL each) for either 45 min or 5 h. Surviving populations are reported as percent change compared to the no nanobead control. Significance values as reported in Figure 3C. Error bars represent the standard error based on three replicates of each experimental test. Significance test results reported above the bars as *p*-values from Student’s *t*-test as follows; ns > 0.05, * < 0.05, ** < 0.01, *** < 0.001.

To determine if PLAN-M nanobeads have potential as a prophylactic treatment on materials such as hospital masks, we investigated their lytic effectiveness in a standard textile protocol. The AATCC-100-2004 antibacterial finishes on textile materials industry-standard assessment is regularly used to evaluate the antibacterial efficacy of textiles (Figure 4A; Pinho et al., 2011). Based on the results obtained from cell exposure in liquid culture, the higher concentration of 80 mg mL^–1^ of tailored PLAN-M nanobeads was applied. Nanobeads without displayed endolysins (PhaC-only) were also included as a negative control.

The AATCC-100-2004 test resulted in significant cell death of the *M. smegmatis* MC^2^ 155 cells after either 45 min of 5 h of exposure. On average it appears that the Lysin A nanobeads were less efficient than the Lysin B nanobeads. After 45 min, exposure to D29-Lysin B resulted in the highest estimated cell lysis with only 18.8% of the population surviving (Figure 4B, grey). The least effective PLAN-M nanobeads in this condition were the two Lysin A endolysins, Pipsqueaks and StarStuff, each of which had a population survival of 68.6 and 68.7%, respectively, leaving over 50% of exposed cells alive in either treatment. After 5 h, the 80 mg mL^–1^ exposure resulted in the highest cell death observed in PLAN-M D29-Lysin B nanobeads with a survival rate of 9.31% (Figure 4B, black). The two Lysin A endolysins performed significantly less well with an average survival of 68%, compared to the Lysin B nanobeads which average survival of 43.5% (*T*-test, *p* = 0.0017). This fabric test was the first time in which a difference between the performance of Lysin A and Lysin B was noted which may have wider implications for their use in PPE.

*M. smegmatis* MC^2^ 155 colonies that survived the AATCC-100-2004 assay may have done so either because they had a pre-existing resistance to the endolysin treatment or because they did not physically interact with the endolysins in a way that resulted in lysis (stochastic survival). In order to test this hypothesis, we took two approaches; the first of these was an attempt to determine if the surviving isolates had an increased resistance to bacteriophage infection by bacteriophage Inca using a standard spot plate dilution as a lawn in a top agar overlay. In every case we tested, these cells appeared as sensitive to bacteriophage infection (as measured by plaque formation) as the naïve *M. smegmatis* MC^2^ 155 (Supplementary Figure 2A).

The second approach we took was to subject cultures of these representative bacterial survivor colonies to a second round of the pure culture assay PLAN-M nanobeads of the same type (Supplementary Figure 2B). None of the bacterial isolates tested appeared to have an increased ability to survive PLAN-M nanobead exposure because of any acquired resistance to endolysin mediated lysis.

The process of a normal infection would include exposure to both the Lysin A and Lysin B enzymes in order to bring about the timely and complete lysis of the *M. smegmatis* cell. We therefore tested if the combination of treatment with both enzymes had a synergistic net effect on overall cell lysis in the liquid assay. We selected the Lysin B from D29 and the Lysin A from StarStuff for a combined lysin test at the 80 mg mL^–1^ condition and measured the relative lysis for these at both 45 min and 5 h. Rather than observing an increase in the effectiveness of these endolysins when used in combination we observed that the cell survival at 45 min was lower at 25.5% than for D29 alone (18.8%) (Figure 4C). At a longer exposure of 5 h the cell lysis of the Lysin A and Lysin B treatment combined was not significantly different from the Lysins used independently and all pair-wise *T*-tests were 0.130 or above in value. This may suggest that combining the activities of Lysin A and Lysin B in this way was not advantageous in the pure liquid culture test employed here.

## Discussion

We tested the ability of these tailored nanobeads to lyse *M. smegmatis* MC^2^ 155 cells in a standing liquid and in a textile surface application and observed an excess of 1 log reduction in cell survival in exposed *M. smegmatis* MC^2^ 155 cells in the presence of PhaC Lysin Attached Nanobeads for Mycobacteria (PLAN-M nanobeads). The ease of production of PLAN-M nanobeads, without the need for experimentally intensive protein purification procedures, opens up a new potential prophylactic intervention of pathogens such as *M. tuberculosis* that can be transmitted by droplets or airborne routes. Tailored nanobeads that have been produced in this manner could be used to limit the risk of infection by aerosol-generated pathogens on objects such as hospital masks, gowns, instruments, air filters and bench surfaces. Such an application would allow us to protect individuals at risk of infection including community members and healthcare workers in the field.

Endolysins are a particularly potent antibacterial because the development of resistance to the action of endolysins is rare (Nelson et al., 2012; Grover et al., 2014; Gerstmans et al., 2016). Previous work demonstrated that endolysins applied extracellularly have not resulted in emerging resistance in *Strepotococcus pneumonia*, *S. pyogenes*, and *Bacillus anthracis* (Fischetti, 2005; Nelson et al., 2012). Our own efforts to determine whether the bacterial cells that survived an initial PLAN-M endolysin-mediated cell lysis indicated that cells that had survived exposure remained sensitive to both bacteriophage infection and PLAN-M nanobeads in repeat exposures (Supplementary Figures 2A,B). Interestingly, resistance to endolysins may be difficult to amass in natural populations because these enzymes are deployed when infected cells lack DNA and therefore have no means by which to transmit resistance to daughter cells (Hyman and Abedon, 2010). Genome degradation in infected cells will prevent direct selection of resistance traits meaning that indirect or weak effects such as kin selection would be required.

The Mycobacteriophage endolysin enzymes described herein Lysin A, a peptidoglycan hydrolase and Lysin B, a lipolytic enzyme have the potential to have a wider host range than the bacteriophages they have been sourced from. Previous work has demonstrated that lytic enzymes functionally displayed on PHA nanobeads can have an expanded range of hosts against which they are active (Altermann et al., 2018).

Tailored nanobeads provide an effective delivery vehicle for these lytic enzymes. The potential to use the lytic enzymes for which existing resistance does not exist suggests that this biotechnology could be a step toward producing safe, economical, environmentally friendly prophylactic surfaces in the battle against bacterial pathogens. PPE is key to protecting the health of essential medical personnel and community members (Hannan et al., 2000; Lee, 2016; Malotle et al., 2017). The ability to manufacture tailored nanobeads like PLAN-M provides an easy and safe way of increasing the effectiveness of PPE to protect against dangerous pathogens.

A set of PLAN-M nanobeads displaying endolysins from mycobacteriophages was designed and tested for their efficacy in lysing *M. smegmatis* MC^2^ 155 cells. These polyhydroxyalkanoate nanobeads can be expressed in a one-step procedure in *E. coli* cells and harvested with minimal post-processing. We functionally fused two Lysin A proteins and six Lysin B proteins to the PhaC synthase protein. Subsequent expression of the fusion protein led to functionalized nanobeads displaying the respective lytic enzyme on the nanobead surface.

The biological activity of these functionalized nanobeads was validated in pure cultures of *M. smegmatis* MC^2^ 155. Control nanobeads (PhaC) displaying no lytic enzymes did not decrease cell viability as measured by efficiency of plating. The Lysin A nanobeads, Pipsqueaks and StarStuff exhibited an expeditious mode of action, decreasing *M. smegmatis* MC^2^ 155 viability by 40–50% within 45 min at a dose of 10 mg/mL. No further decline in viability was observed after 8 h. Lysin A enzymes are peptidoglycan hydrolyzing enzymes. We hypothesize that these Lysin A enzymes do not release the peptidoglycan moiety to which they are bound, effectively inactivating the enzyme after one enzymatic action. This would lead to an increasingly diminished activity of Lysin A nanobeads, ultimately resulting in a static state. This hypothesis is supported by the effects observed at the higher dose (80 mg/mL), an initial decline in viability that does not decrease further over time.

Lysin B enzymes weaken cells by cleaving the bonds between the arabinogalactan layer and the outer mycolic acids. Although the reason that this leads to cell death is not currently well understood, the effectiveness of Lysin B enzymes to mediate lysis from without is well documented Payne (2012). The degree of lytic activity observed in the Lysin B nanobeads varied considerably. In contrast to the Lysin A activity at 45 min (10 m/mL) the Lysin B nanobeads ranged from 20.0 to 70.0% cell lysis. Two patterns arose in the Lysin B activity after 5 h. D29, Giles and Inca all resulted in a further reduction in viability whereas Bongo, JAWS and Dylan saw a recovery of *M. smegmatis* MC^2^ 155 populations. This may indicate that the biological activity of the Lysin B enzymes, at this low concentration, was sufficient to damage cells but that some recovery was possible after 5 h. Conversely, at higher concentrations cell viability was decreased for all enzymes at all-time points. Notably, D29 and Inca exhibited the greatest activities at low and high concentrations at 5 h, respectively, making them the most promising candidates for fabric testing.

Having established that all of the functional nanobeads displaying Lysin A and Lysin B have biological activity against *M. smegmatis* MC^2^ 155 in pure culture, we turned our attention to the development of fabric facemasks, or other PPE, that feature anti-*Mycobacterium* activity mediated by functionalized nanobeads. Commercially available facemasks or PPE are highly hydrophobic. We therefore propose to incorporate functionalized nanobeads as a central fabric barrier, sandwiched between the outer layers.

One of the known transmission routes of *M. tuberculosis* and other pathogens is through airborne droplets. We therefore adapted the AATCC-100-2004 protocol for testing the biocidal character of treated textiles (American Association of Textile Chemists and Colorists, 2005). We focused on the higher concentration of nanobeads for these assays in order to account for anticipated loss of efficacy due to occlusion effects in fabric matrix.

As expected, the efficacies for nanobeads immobilized in fabrics were less pronounced than in pure cultures. An average population decrease of 30% was observed with nanobeads displaying either of the Lysin A enzymes, Pipsqueaks or StarStuff. Similar to the results seen in the liquid culture assay cell death was detected after 45 min and no culture recovery was observed after 5 h. As anticipated, the activity observed was approximately half as biocidal as the same enzymes in the pure culture assay.

The nanobeads displaying the Lysin B enzymes generally performed worse in the textile assays than they did in the pure culture assay with a loss of efficacy of roughly 20.45%. In the case of Giles, cell lysis was less efficient at 45 min (54% lysis) compared to the pure culture (70% lysis). This trend was reversed at 5 h, where 70% cell lysis was achieved on textiles compared to pure culture (71%). In contrast, nanobeads displaying the D29 Lysin B performed better on textiles than in pure culture. Here we observed a statistically significant 15% increase in efficacy in the textile matrix compared to the pure culture at 45 min, an increase from 69 to 81%. The decrease in cell viability continued at 5 h reaching a 91% reduction in cell viability representing a 25% increase in efficacy compared to pure culture at 68%. It has been suggested that the lytic activity of the D29 Lysin B comes from cleaving the ester bond at the mycolylarabinogactan interface in the Mycobacterial cell wall and that this detachment makes the cells susceptible to osmotic shock (Sharma et al., 2018). It is possible that the presence of a surface in this assay exacerbates this effect further.

We have observed up to a 1 log fold decrease in Mycobacterial populations due to exposure to nanobeads displaying functional nanobeads our biocidal textile assay. This represents a significant reduction in potentially infectious particles in the PPE application that we are envisioning in which between 20,000 and 700 infectious particles can be transmitted in a droplet by a sneeze or a cough, respectively (Fernstrom and Goldblatt, 2013).

Droplet based pathogen spread is currently a significant issue in these settings where protecting the health of patients, community members and medical staff are of paramount importance (Jones et al., 2020).

We are currently planning to investigate the efficacy of prototype PPE (i.e., Masks) with functionalized nanobead enhancements in clinical model systems. A promising avenue for future work would be to consider the combined effects of the lysins described here. Lysin A kills the cells but may not be as efficient as Lysin B. Dual Fusion nanobeads that display both on the same PhaC enzyme will enable us to test the true synergy of these enzymes against *M. smegmatis* MC^2^155.

In addition, we note that bacteriophage D29, has been used to infect *M. ulcerans* (Fraga et al., 2019) and it infects *M. tuberculosis* both *in vitro* and in a mouse model (Carrigy et al., 2019; Bavda and Jain, 2020). In light of these results and in combination with our present data suggesting that the Lysin B of D29 is both fast -acting and stable as part of a functionalized nanobead, this Lysin B is an extremely attractive candidate for a bespoke nanobead PPE application that would protect against *M. tuberculosis* pathogens.

## Data Availability Statement

The raw data supporting the conclusions of this article will be made available by the authors, without undue reservation.

## Author Contributions

EA and HH conceived of the project. CD carried out all experiments, collected results, and chose the endolysins and performed bioinformatics. EA designed the constructs for producing PLAN-M nanobeads. KR was instrumental in teaching CD how to produce and use PLAN-M nanobeads. HH and CD wrote the final manuscript and prepared all final figures. All authors read and approved of the final manuscript.

## Conflict of Interest

EA and KR were employed by the company AgResearch. The remaining authors declare that the research was conducted in the absence of any commercial or financial relationships that could be construed as a potential conflict of interest.
