# Supplementary material for: PLAN-M; Mycobacteriophage Endolysins Fused to Biodegradable Nanobeads Mitigate Mycobacterial Growth in Liquid and on Surfaces
Source: Front Microbiol. 2021 Apr 26;12:562748. doi: 10.3389/fmicb.2021.562748 (PMC8107382; doi:10.3389/fmicb.2021.562748)
Supplement: Supplementary file 1 [file Data_Sheet_1.docx]

**Supplementary Figure and Figure Legend**

**Supplementary Figure 1**

**
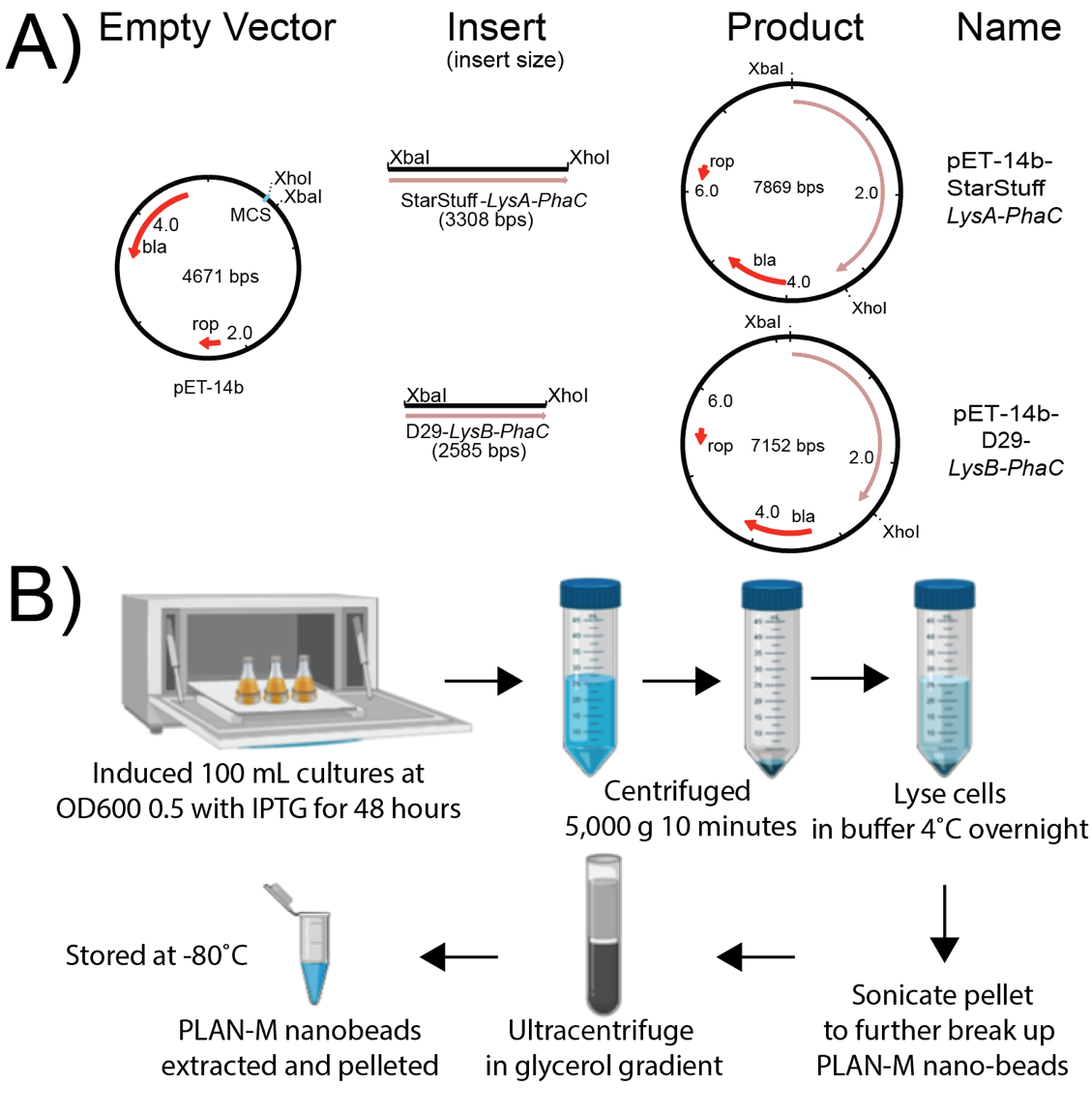
**

**Supplementary Figure 1.** A) Diagram of cloning process. The pET-14b vector expression system enables the high-level expression of the selected protein, functionally fused to PhaC, which will produce the PHA polymer. The empty vector (left) includes a lacI repressor, a lacO operator, a promoter that is specific for the T7 RNA polymerase upstream of the MCS, (Bla) Ampicillin resistance gene and an origin of replication. Representative bacteriophage-Lysin-PhaC fusion inserts are shown for StarStuff-LysA and the D29-LysB. The final constructs and the

respective names are shown at right. B) An overview of the synthesis process for creating PLAN-M

nanobeads (see materials and methods).

**Supplementary Figure 2**

**
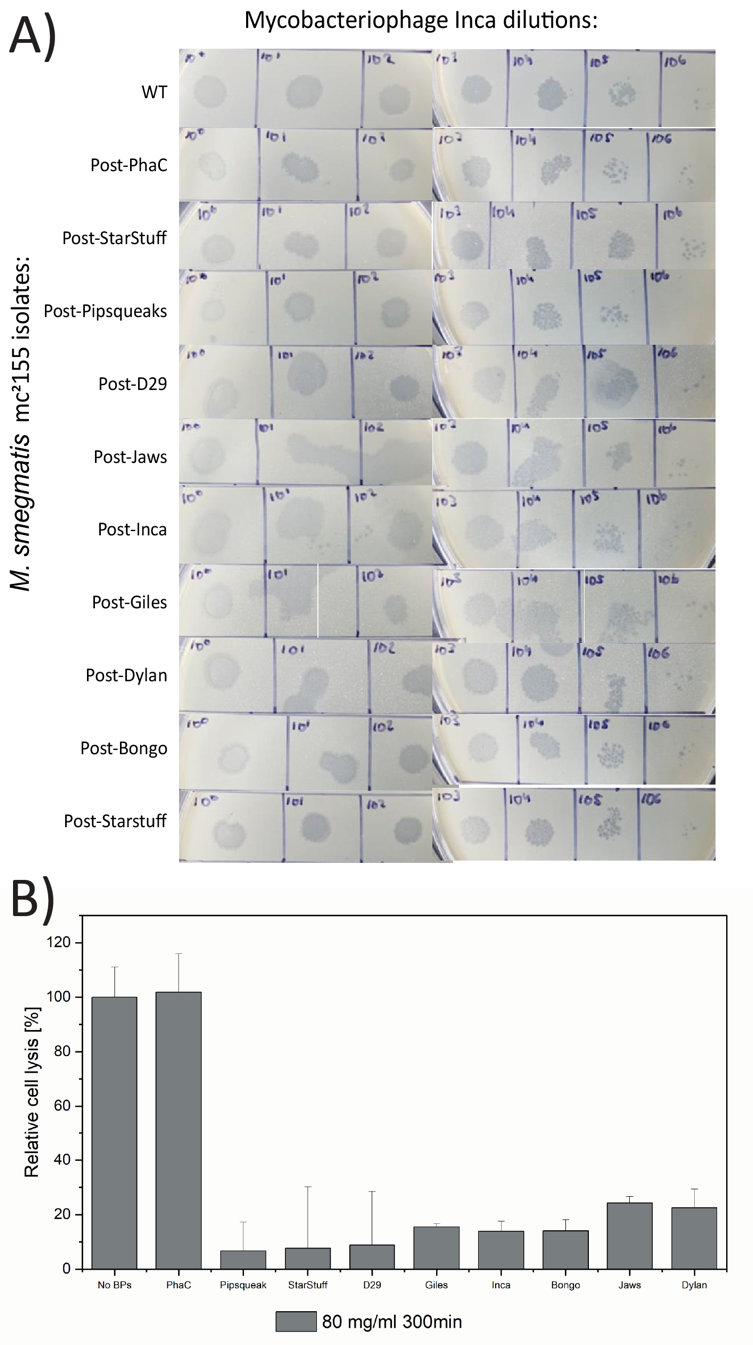
**

**Supplementary Figure 2. Evaluating Endolysin Resistance in Surviving Colonies.** Bacteria from the liquid culture CFU plates that had survived PLAN-M nanobead exposure were preserved and used in A) A serial dilution plaque formation assay using bacteriophage Inca. B) These cultures were also added to a pure culture CFU assay once again as shown in Figure 3A. The results of the population lysis from the pure culture assay showed that these cells were not more resistant than the naïve WT *M. smegmatis* MC^2^ 155 cells (N=3), error bars show standard error for samples.
